# Phase-of-firing coding of dynamical whisker stimuli and the thalamocortical code in barrel cortex

**DOI:** 10.1186/1471-2202-14-S1-P279

**Published:** 2013-07-08

**Authors:** Sohail Siadatnejad, Michael Bale, Rasmus S Petersen, Marcelo A Montemurro

**Affiliations:** 1 Faculty of Life Sciences, University of Manchester, Manchester, M13 9PL, UK

## 

**Figure 1:**
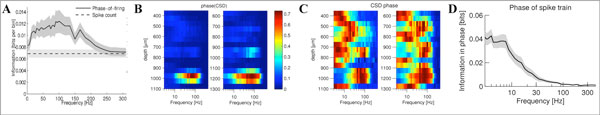
**A. *Phase-of-firing *information and and spike rate information averaged across channels**. **B**. Depth-frequency profile of the CSD phase information across the cortical layers (for two electrodes in two animals). **C**. same as B, but normalised to reveal the bands with peak information. **D**. Time-resolution of the spike responses.

In the *phase-of-firing *(PoF) code the information carried by spikes is boosted when their firing is measured against the angular phase of the local extracellular signal. This coding strategy has been verified so far in visual and auditory cortices of monkeys at low frequencies [1,2]. Here we present the first evidence of this code in whisker modality. In particular, we characterise the PoF encoding of the white-noise whisker deflections in the barrel cortex responses of urethane anaesthetized rats. A novel aspect of our results is identifying the high frequency components of the PoF responses at certain cortical layers, with implications regarding the thalamo-cortical code and cortical information processing. The results indicate that the amount of information encoded using a PoF code was on average 100% greater than a spike rate code using MUA (Figure 1A), and this was up to 250% in deeper channels of the cortical columns. Contrary to the previous findings [1,2], the extra information in PoF using LFP was peaked at very high frequencies (100 Hz). The effect extended to >200 Hz bands of LFP. When CSD was used for labelling spikes, the effect was more localized with respect to depth, and was peaked at different frequencies at different cortical layers (Figure 1C). The depth-frequency profile was consistent across the multi-channel electrode penetrations. Why the PoF information was maximal at such high-frequency bands? ( >100 Hz) We hypothesised that the high-frequency PoF components originated from the high-frequency components in the white-noise stimuli. The depth-frequency profile and CSD analysis of layers showed association of the high-frequency components of PoF with the cortical layers that are known to receive direct thalamo-cortical input from VPM. A similar pattern was previously suggested in [5]. The high-frequency components of CSD phase responses were weak or absent in layers that are not associated with such projections (Figure 1C).

We then examined whether the pure spiking activity contributes in the information manifested in the high frequency PoF components. The temporal precision of spikes was quantified using a novel measure, information in the phase of the band-bass filtered spike trains. In all depths, the spikes lacked any temporal-resolution larger than 30 Hz (Figure 1D). We conclude that the output of the local computational processes in a cortical column lacked the temporal precision that they received in input (i.e., which was represented by CSD). This suggests a transformation of the information encoding from a high-precision input code relayed from thalamus, with fast and temporally precise dynamics (see [4]), into an output spiking code with a lower temporal resolution. Nevertheless, information about the high-frequency and fast varying components of the stimulus may still be encoded within the low resolution and irregular cortical spikes. In fact previous studies have shown that a spike-count code in the cortex can encode such stimulus features [3]. The computational demands of the whisker system (such as fine texture discrimination, and perception of subtle vibrations in the air) require that the thalamus relays the high-frequency components of the whisker deflections into the cortical barrels, which are capable of performing more complex neural computation.
